# Iatrogenic metacarpal fracture after K-wire fixation: A case report and prevention

**DOI:** 10.1016/j.ijscr.2019.11.003

**Published:** 2019-11-09

**Authors:** Ja Hea Gu, Su Hyun Choi

**Affiliations:** Department of Plastic Surgery, Dankook University Hospital, Cheonan, Chungnam, South Korea

**Keywords:** Iatrogenic fracture, K-wire fixation, CMC subluxation, Metacarpal base fracture, Complication, Case report

## Abstract

**Introduction:**

Iatrogenic fractures after failed K-wire fixation in the management of a carpometacarpal (CMC) joint fracture-dislocation have not yet been reported (Hsu et al., 2011). We present a case of K-wire-related complication in the management of a CMC joint fracture-dislocation and highlight the importance of planning K-wire placement and minimizing the number of K-wire passes.

**Presentation of case:**

After beating his hand against a wall, a 22-year-old patient visited our clinic complaining of a swollen and painful wrist. Following our protocol, reduction and K-wire fixation was planned. During these procedures, the resident of our team made several attempts to insert transfixation pins and radiologic finding demonstrated incorrect placement of the K-wire track. The patient visited the outpatient clinic at 5 weeks postoperatively then we removed the K-wires and began mobilization. Two weeks later, the patient came back with acute onset of pain and swelling at the 5th metacarpal area.

**Discussion:**

Stahl and Schwartz reported that 27.8 % of complications related K-wire were due to technical failure and 90 % of technical failure were caused by hospital residents. Well-established guidelines and supervision by a highly experienced surgeon is likely to reduce the rate of technical failure. Multiple passes of the K-wire have resulted in blunting of the K-wire and subsequent heat generation then lead to subsequent loosening and loss of fixation.

**Conclusion:**

Preoperative planning, marking the K-wire route, and appropriate K-wire thickness minimize such complications. Patients should be informed that following K-wire removal, the residual holes could be subject to stress risers.

## Highlights

• There are possibility of iatragenic fracture anywhere K-wire have passed and failed.
• Surgeons should be aware of the potential damage to bone during K-wire fixation.
• Preoperative planning, continuous C-arm scanning and appropriate K-wire thickness can minimize complications.
• Patients should be told that following K-wire removal, the residual holes could subject to stress risers.

## 1 Introduction

For unstable fracture-dislocations of the fifth Carpometacarpal (CMC) joint, authors prefer closed reduction and percutaneous pinning. Pin fixation methods are simple and reliable in fracture fixation however, complications associated with the Kirschner wire (K-wire) procedure are various as follows: osteomyelitis, tendon rupture, nerve lesion, pin tract infection, pin loosening, migration and fractures through pin site.

Hsu et al. [1] reported 1 case of fracture through pin track out of 408 K-wire fixations on the hand and wrist. Loh et al. [2] reported one more case of iatrogenic Bennett fracture complication secondary to K-wire fixation. However, from out literature iatrogenic fractures after failed K-wire fixation site in the management of a CMC joint fracture-dislocation have not yet been reported up to this point and almost all cases of iatrogenic fractures are developed in fracture site.

We present a K-wire-related complication in the management of a CMC joint fracture-dislocation and would like to highlight the importance of planning K-wire placement and minimizing the number of K-wire passes.

## 2 Presentation of case

**Fig. 1:**
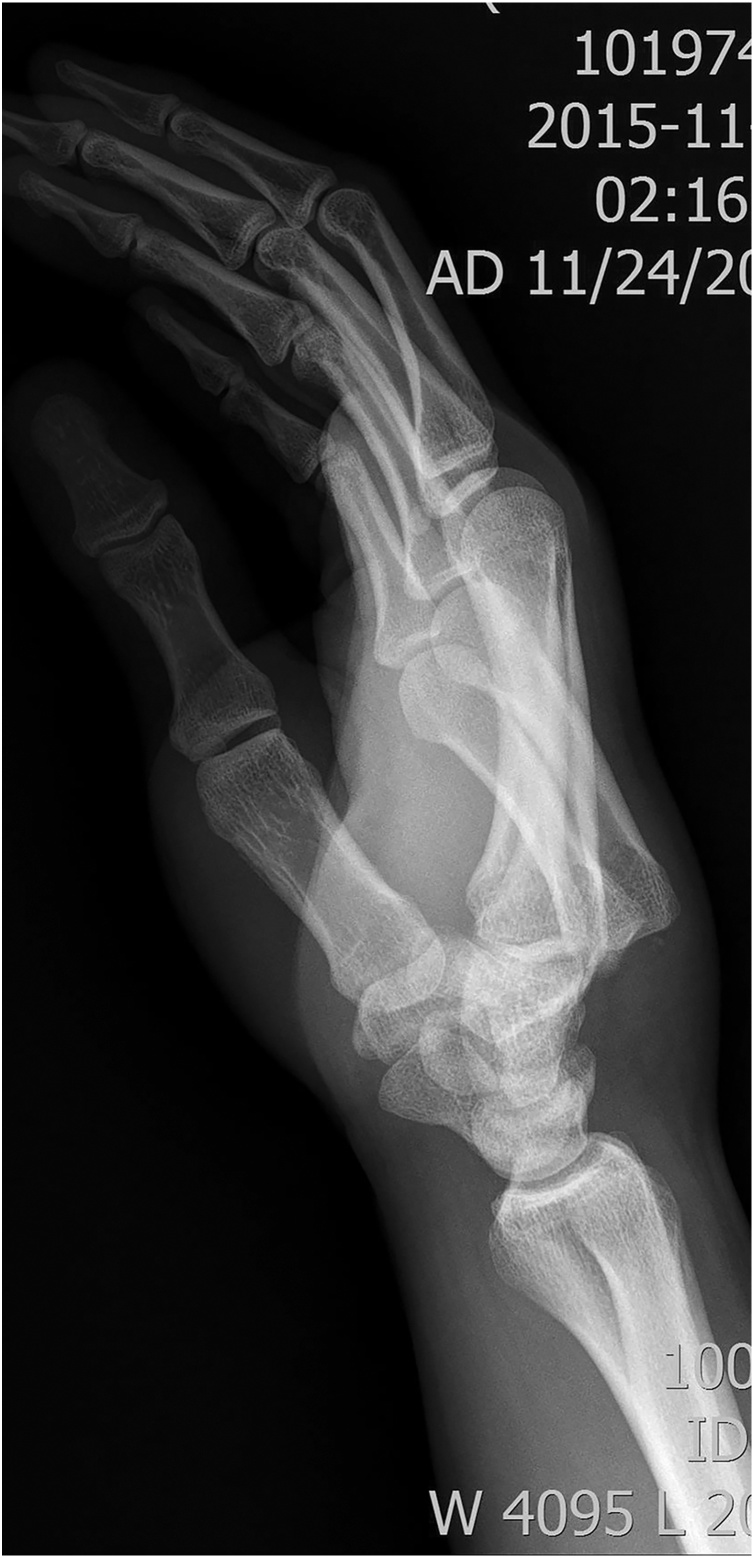
Pre-operative X-ray (lateral view). X-ray demonstrate a posterior dislocation of the 4th and 5th metacarpal bases in CMC joint, with fractured tiny bone fragments in the ventral side of the CMC joint.

**Fig. 2:**
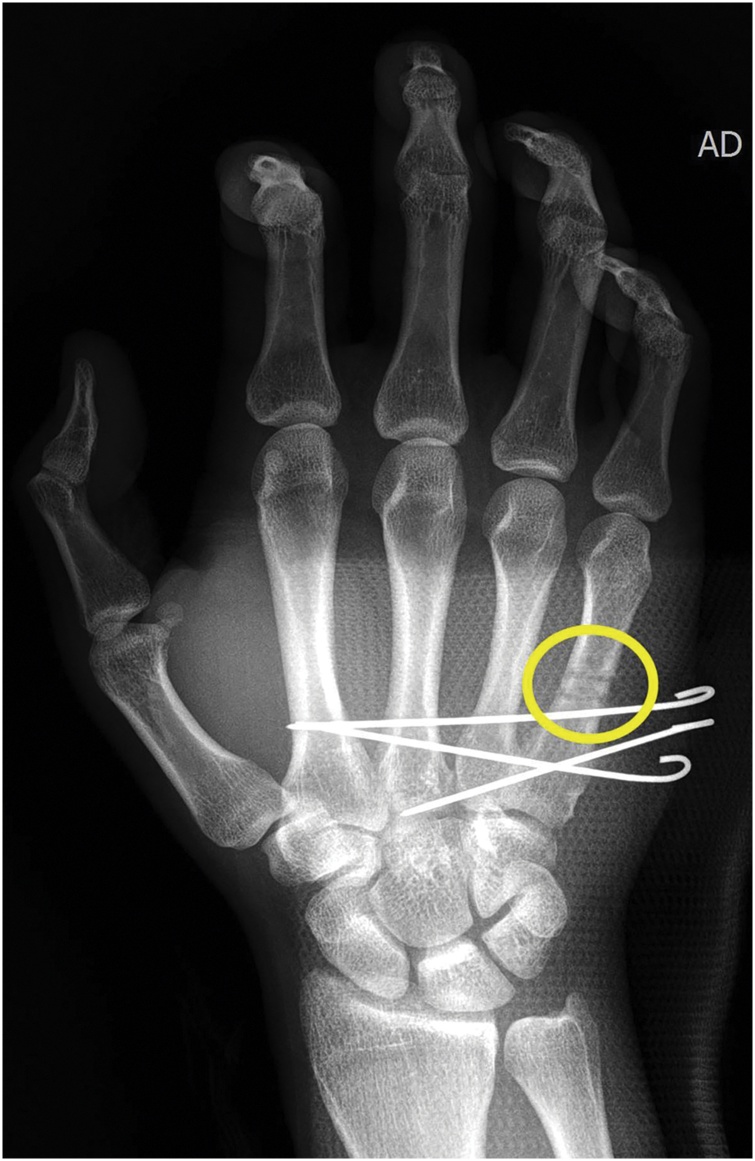
Intra-operative, immediate post-operative X-ray. Although closed reduction was attempted, the CMC joints were unstable. Following our protocol, reduction was obtained by longitudinal traction and lateral pressure on the displaced bone and fixation with a transarticular pin and transfixation pins into the adjacent metacarpal was planned. During these procedures, the resident of our team made several attempts to insert transfixation pins with 1.1 mm K-wire into the metacarpal shaft, but each attempt failed. After final fixation, immediate postoperative X-ray showed the K-wire tracks (Yellow Circle).

**Fig. 3:**
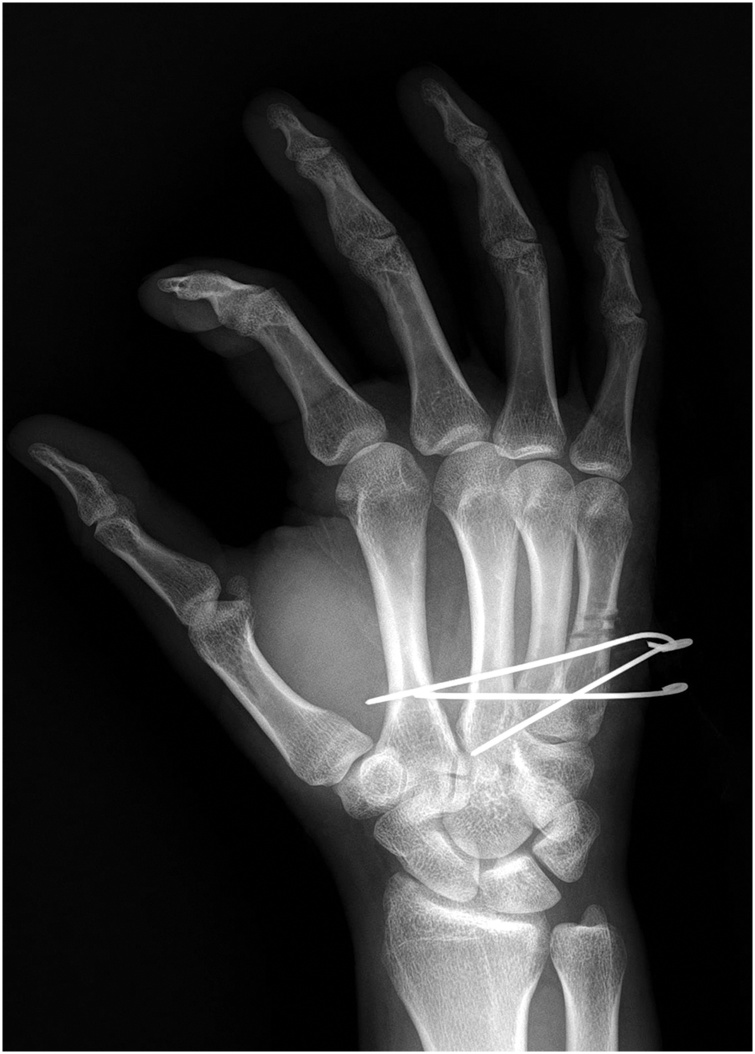
Simple X-ray at five weeks postoperatively. Simple X-ray of his hand demonstrated the union of the fractured site but the K-wire track still showed with no signs of displacement.

**Fig. 4:**
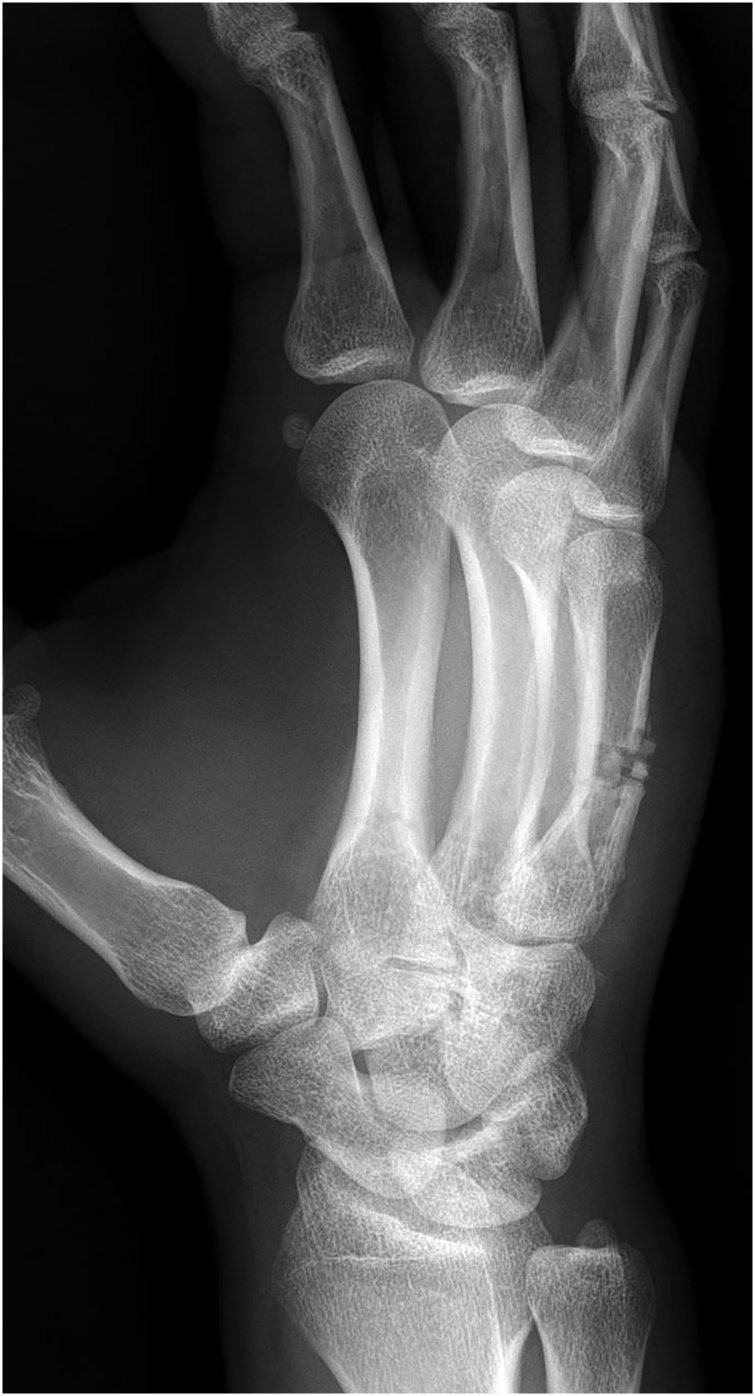
Iatrogenic fracture developed following failed K-wire tracks. Two weeks later, the patient came back with acute onset of pain and swelling at the 5^th^ metacarpal area. A radiograph of his hand was performed, which demonstrated a fracture through the metacarpal shaft where the K-wire had failed to enter the bone.

**Fig. 5:**
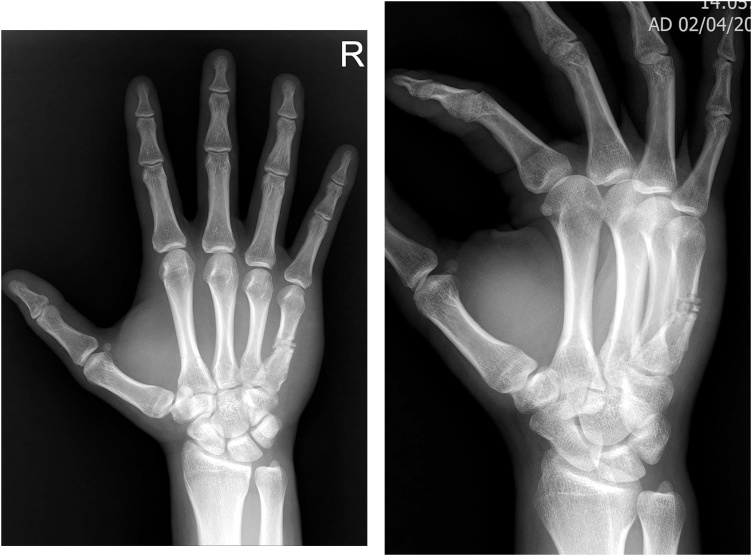
Simple X-ray (AP view (left) and oblique view (right) Postoperative 8 weeks. Initial CMC dislocation and fracture was well healed, however displacement of fracture on metacarpal body caused by secondary fracture is aggravated and malunion was suspected.

Our study was reported following SCARE guidelines [3]. After having beaten against the wall, a 22-year-old patient was visited our clinic complaining of swollen and painful wrist. He did not have any kinds of drug history, family history including any relevant genetic information, and psychosocial history. Computed tomography (CT) and X-ray was performed and diagnosed as posterior dislocation of 4^th^ and 5^th^ metacarpal bases in the carpometacarpal joints (CMCJ) and fractured tiny bone fragments are found in the ventral side of CMC joint (Fig. 1). Although closed reduction was tried, CMC joints were unstable. For unstable fracture-dislocations of the CMC joints, we prefer closed reduction and percutaneous pinning. Following our protocol, reduction was obtained by longitudinal traction and lateral pressure on displaced bone and fixation with a transarticular pin and transfixation pins into the adjacent metacarpal was planned to allow early motion. During these procedures, the senior resident of our team made several attempts to insert transfixation pins with 1.1 mm K-wire into the metacarpal shaft but, failed. Radiologic finding demonstrated incorrect placement of the K-wire track (Fig. 2), however we think it will be healed and union with splinting. We neglected the K-wire tract and just follow our usual protocol. Patients are tolerable during surgery. He discharged on next day of surgery with short arm splint. The patient visited the outpatient clinic at 5 weeks postoperatively, and then underwent a radiologic exam. Simple X-ray of his hand demonstrated the union of the fractured site. Although the K-wire track still showed, there were no signs of displacement (Fig. 3). We removed the K-wires and began mobilization. Two weeks later, the patient came back with acute onset of pain and swelling without any trauma experience at the 5th metacarpal area. A radiograph of his hand was performed, which demonstrated a fracture through the metacarpal shaft where the K-wire had failed to enter the bone (Fig. 4). Patients didn’t want to more surgery then, he was treated with an ulnar gutter splint for the second fracture. He made an uneventful recovery postoperative 8 weeks. However, displacement is aggravated and malunion is suspected (Fig. 5). We recommend further surgery such as bone graft, however he refused and did not show at his next reservation (6 months after operation).

## 3 Discussion

While K-wire fixation is a common and versatile method of internal fixation of fractures to arthrodesis in the hand and wrist, 15 %–18 % of complications are reported from minor to major [1,[4], [5], [6]]. Besides one of article [7] reported up to 42 percent of complications related K-wire fixation on distal interphalangeal joint site. Stahl and Schwartz [4] reported that 27.8 % of complications related K-wire were due to technical failure and 90 % of technical failure were caused by hospital residents. Adherence to well-established guidelines and supervision by a highly experienced surgeon is likely to reduce the rate of technical failure [5]. However, other article for pediatric cases [4] demonstrated that the complication related K-wire was depend on not only surgeon’s technique but the amount of fracture. Multiple passes of the K-wire onto cortical and cancellous bone, resulting in blunting of the K-wire and subsequent heat generation. A zone of necrosis around the pin site can lead to subsequent loosening and loss of fixation. In our case, I think we neglected avascular necrosis of bony fragment between failed K-wire tracks. These fragments were absorbed after a few weeks then the fracture was not union but developed fracture. Surgeons should be aware of the potential damage to bone during K-wire fixation. Although metacarpal bone is relatively thick bone in hand, there are also possibility of iatrogenic fracture after failed fixation and iatrogenic fracture can be developed anywhere K-wire have passed. Our patients had two failed track marks therefore, I should consider a zone of necrosis could be made between two failed tracks. For this kind of case, malunion could be developed and might need further surgery including bone graft. Surgeons also should be careful when they fix to the adjacent bone such as treating CMC or carpal bone fracture. During follow up period, if there are multiple trial during operation, longer duration of immobilization and close observation needs to be considered.

## 4 Conclusion

Preoperative planning, marking the K-wire route on the skin and appropriate K-wire thickness minimize complications and during operation continuous C-arm monitoring is also helpful. As a supervisor, we should emphasize to beginner how risky to try multiple attempts during K-wire fixation. Patients should be informed that following K-wire removal, the residual holes could subject to stress risers and that they will not be able to use their injured hand normally for several weeks.

To minimize possibility of iatrogenic fracture, using continuous C-arm monitoring to reduce attempts of K-wire fixation and supervising on-training surgeons strictly are important.

## Sources of funding

No funding was received for this article.

## Ethical approval

Not applicable. (one case who underwent usual treatment, we got a patient consent and agreement for publication).

## Consent

Written informed consent was obtained from the patient for publication of this case report and accompanying images. A copy of the written consent is available for review by the Editor-in-Chief of this journal on request.

## Author’s contribution

Ja Hea Gu: Study concept or design, Data analysis and interpretation, Confirmation of final paper.

Su Hyun Choi: Writing the draft, data collection.

## Registration of research studies

This article is not human studies. Report of one case who underwent usual treatment.

## Guarantor

Ja Hea Gu has a full responsibility for the work and the conduct of the study, access to the data and controlled the decision to publish.

## Provenance and peer review

Not commissioned, externally peer-reviewed.

## Declaration of Competing Interest

The authors have nothing to disclose.
